# Does Variation in Genome Sizes Reflect Adaptive or Neutral Processes? New Clues from *Passiflora*

**DOI:** 10.1371/journal.pone.0018212

**Published:** 2011-03-28

**Authors:** Karla S. C. Yotoko, Marcelo C. Dornelas, Pakisa D. Togni, Tamara C. Fonseca, Francisco M. Salzano, Sandro L. Bonatto, Loreta B. Freitas

**Affiliations:** 1 Laboratório de Bioinformática e Evolução, Departamento de Biologia Geral, Universidade Federal de Viçosa, Viçosa, Brazil; 2 Departamento de Biologia Vegetal, Instituto de Biologia, Universidade Estadual de Campinas, Campinas, Brazil; 3 Laboratório de Evolução Molecular, Departamento de Genética, Universidade Federal do Rio Grande do Sul, Porto Alegre, Brazil; 4 Laboratório de Biologia Genômica e Molecular, Faculdade de Biociências, Pontifícia Universidade Católica do Rio Grande do Sul, Porto Alegre, Brazil; Instituto de Biología Molecular y Celular de Plantas, Spain

## Abstract

One of the long-standing paradoxes in genomic evolution is the observation that much of the genome is composed of repetitive DNA which has been typically regarded as superfluous to the function of the genome in generating phenotypes. In this work, we used comparative phylogenetic approaches to investigate if the variations in genome sizes (GS) should be considered as adaptive or neutral processes by the comparison between GS and flower diameters (FD) of 50 *Passiflora* species, more specifically, within its two most species-rich subgenera, *Passiflora* and *Decaloba*. For this, we have constructed a phylogenetic tree of these species, estimated GS and FD of them, inferred the tempo and mode of evolution of these traits and their correlations, using both current and phylogenetically independent contrasted values. We found significant correlations among the traits, when considering the complete set of data or only the subgenus *Passiflora*, whereas no correlations were observed within *Decaloba*. Herein, we present convincing evidence of adaptive evolution of GS, as well as clues that this pattern is limited by a minimum genome size, which could reduce both the possibilities of changes in GS and the possibility of phenotypic responses to environment changes.

## Introduction

The C-value paradox [1]–[3], or the lack of correlation between genome sizes (GS) and number of genes and organism complexity is a well-known phenomenon. Within plants, there is more than a 2000-fold variation in GS [4], which may varies considerably even between very closely related species. This variation is ultimately produced by mutational mechanisms, which include unequal chromosome crossover [5], DNA replication errors [6]–[8], polyploidization [9], [10], gene duplication [11] and the proliferation of transposable elements [12]–[14]. In relation to the latter, some plants present more than 60% of their genomes comprised of transposable elements [15].

An open question regarding such GS variation concerns the mechanisms that maintain extra DNA within species. Some theories propose a neutral evolution of genome sizes: *i.* Junk DNA theories propose that extra DNA, considered useless and maladaptive, is fixed by random drift and carried passively in the chromosomes, since purifying selection against it is not strong enough [16]–[17]. According to this view, extra DNA would increase until the highest tolerable maximum, which would depend on the specific organism ecological and developmental needs. *ii.* The mutational equilibrium model [18], on the other hand, suggests that a balance between the DNA loss occurring through the predominance of small deletions over small insertions and the DNA gain obtained through the predominance of large insertions over large deletions, determine the equilibrium of GS. *iii.* The proportional model of GS evolution [19] uses a probabilistic approach to suggest that the rate of genome size evolution is proportional to the size of the genome in question, with faster rates occurring in the larger genomes. Therefore, according to this view, it would be more difficult for small genomes to become and stay larger and easier for large genomes to become and stay smaller, explaining why (regardless of the GS variation range within eukaryotes), the GS of most species tends to be short [20].

On the other hand, there are some evidences for genome size adaptive evolution coming from the correlation between GS and various phenotypic traits of apparent selective significance, such as seed size [21], [22], response of annual plants to CO_2_ [23], metabolic rates [24]–[27], recombination rates [28], seedling development [29], flower size [30], [31], among others. As for environmental characters, Knight and Ackerly [32] found correlation between GS and extreme temperatures or annual precipitations and Achigan-Dako and colleagues [33] found a correlation between GS and altitude for *Lagenaria siceraria*. On the other hand, Knight and Beaulieu [34] suggested that genome size correlations are quite strong at the cellular level but weak in predictive power with increasing phenotypic scale. Indeed, Bennet [35] proposed the well-known nucleotype effect, or positive correlation between GS and nucleus size, or between cell size and duration of mitosis and meiosis, suggesting that DNA content is associated with life history traits, once annuals have smaller GS than perennials [35]–[38].

In order to try to understand the tempo and mode of GS evolution, we considered in this work the genome size evolution within the genus *Passiflora* and, more specifically, within its two most species-rich subgenera [39]: *Passiflora* (240 spp) and *Decaloba* (235 spp). Although being sister clades [40], [41], *Passiflora* and *Decaloba* present some ecological, morphological and evolutionary differences. Preliminary data showed that GS sizes between *Decaloba* and *Passiflora* were remarkably different. Thus, we have estimated genome sizes (GS) and flower diameters (FD) of 49 species belonging to *Passiflora* and *Decaloba* subgenera and constructed a phylogenetic hypothesis for these species based on the four most used plastid sequences

Using these data, we have investigated the tempo and mode of evolution of these traits and searched for possible correlations among them. From these results, we have hypothesized evolutionary patterns and processes which could explain the GS evolution within these subgenera.

## Materials and Methods

### Plant material

Table 1 lists the 50 *Passiflora* species studied in the present investigation. Thirty six of them are from the subgenus *Passiflora* and 13 are from the subgenus *Decaloba*. *Passiflora deidamioides* from the *Deidamioides* subgenus, was used as outgroup. *Decaloba* occurs in the Americas, but also in Southeast Asia and Australia, and *Passiflora* is restricted to the Americas, ranging from the south of the United States to South America. Species of *Decaloba* are mostly herbaceous vines with small flowers and fruits. Conversely, species in the *Passiflora* subgenus are woody vines with showy flowers and medium to large edible fruits [41]. Regarding the chromosome numbers, most *Decaloba* species present n = 12 (except for *P. suberosa*, 2n = 24), while most *Passiflora* species present 2n = 18 (except for *P. foetida*, 2n = 10).

**Table 1:** *Passiflora* genome sizes and flower diameters.

Species	GS (pg)	FD (cm)	Species	GS (pg)	FD (cm)
Subgenus *Passiflora*			Subgenus *Passiflora*		
*actinia*	1.057	6.33	*pilosicorona*	1.400	10.95
*alata*	2.208	12.52	*racemosa*	1.076	8.73
*caerulea*	1.386	6.37	*serratodigitata*	1.387	8.3
*campanulata*	1.195	6.62	*sidaefolia*	0.928	6.04
*caparidifolia*	2.051	12.8	*subrotunda*	1.318	4.99
*cerasina*	1.319	7.53	*urubiscencis*	1.582	6.27
*coccinea*	1.337	10.11	*vitifolia*	1.414	11.87
*edmundoii*	0.760	6.32	*watsoniana*	1.305	6.14
*edulis*	1.258	6.94	***Passiflora*** ** Average (SD)**	**1.311 (0.431)**	**7.28 (2.23)**
*eischleriana*	1.212	6.57	**Subgenus ** ***Decaloba***		
*foetida*	0.481	3.49	*auriculata*	0.993	2.86
*galbana*	1.386	7.13	*capsularis*	0.319	2.86
*gardinerii*	1.918	7.03	*leptoclada*	0.261	2.75
*gibertii*	1.710	6.82	*micropelata*	0.250	3.94
*hatschbachi*	0.881	6.89	*misera*	0.253	2.72
*incarnata*	0.659	6.9	*morifolia*	0.505	2.91
*iodocarpa*	1.299	7.18	*organensis*	0.212	2.68
*ischnoclada*	0.901	5.51	*pohlii*	0.299	2.5
*jilekii*	0.933	3.93	*suberosa*	0.684	1.42
*kermesina*	1.237	7.81	*tricuspis*	0.287	2.7
*ligularis*	1.414	6.36	*truncata*	0.704	2.48
*loefgrenii*	1.310	6.34	*tulae*	0.277	4.41
*miersii*	1.452	6.46	*vespertilio*	0.327	3.76
*mucronata*	1.512	7.1	***Decaloba*** ** Average (SD)**	**0.413 (0.239)**	**2.92 (0,75)**
*nitida*	1.849	10.39			
*palmeri*	0.263	3.81	**Total Avg (SD)**	**1.073 (0.557)**	**6.12 (2.75)**
*picturata*	2.172	8.02	**OutGroup**		
*platyloba*	1.643	5.53	*deidamioides*	0.815	4.69

Averaged genome sizes (GS, expressed in 1C) and flower diameter (FD) of the *Passiflora* species included in this work.

The samples were obtained from the *Passiflora* Germplasm Collection, Biology Institute, State University of Campinas (IB/UNICAMP), Campinas, SP, Brazil. *Arabidopsis thaliana* Landsberg ecotype seeds, obtained from the ABRC Stock Centre/Ohio State University (Columbus, USA), were germinated in soil and cultivated in growth chambers at 21°C under short day conditions.

### Flow cytometry

About one square inch of fresh young leaf tissue was chopped with a scalpel in 0.5 ml of ice-cold ‘OttoV’ solution (0.1 M citric acid monohydrate, 0.5% v/v Tween 20, [42]) in a disposable sterile Petri dish. The obtained suspension was filtered through a 42 µm nylon mesh and stored frozen at −20°C until use. Two volumes of ‘Otto II’ solution (0.4 M Na_2_HP0_4_.12H_2_0 with 2 µl/ml β-mercaptoethanol, [42]) containing propidium iodide and RNase (each at a final concentration of 50 µg/ml) were added to the thawed samples (at 23–25°C) just before analysis. Sample measurements were run on a Becton-Dickinson FACSCalibur flow cytometer with an argon laser exciting at 488 nm. Pulse area was detected using FL2-A (585 mean/42 bandwidth) with a threshold at FLS 35. Half of the volume of the samples consisted of *Arabidopsis* nuclear suspension, used as an internal standard. The genome size of each sample was calculated using the mean diploid (2C) genome size of the *Arabidopsis* Landsberg ecotype, estimated to be 0.32 pg [43], for comparison.

Total fluorescence, together with pulse height and width fluorescence emitted from the nuclei were collected through a 645-dichroic and a 620-band-pass filter, and converted on 1,024 ADC channels. Prior to analysis the instrument was checked for linearity and the amplification adjusted so that the peak corresponding to 2C *Arabidopsis* nuclei was positioned approximately at channel 200. This setting varied according to the mean DNA content of the species analysed. In some cases we have set 4C or 8C *Arabidopsis* nuclei at channel 200 to accommodate the peak mean of the test-species with larger genomes within the graph frames. In these cases additional cross-tests with other known large-genome species (i.e. *Oriza sativa* and *Solanum lycopersicon*, nuclei prepared as for *Arabidopsis*) were performed, to check for the consistency of the results. Three graphs were obtained: linear-fluorescence light intensity (FL); forward angle (FS) - versus side angle (SS) - light scatter; and FL total pulse versus FL pulse height. The last cytogram was used to eliminate partial nuclei and other debris, nuclei with associated cytoplasm and doublets [44]. A gate area was defined such that only single intact nuclei were included in the FL histogram. We compared the position of the G_0_/G_1_ peak of the sample on a histogram with that of the internal reference plant with known nuclear DNA content (*Arabidopsis*). For each sample at least 10,000 nuclei were analysed. The size of the nuclear genome of each sample was calculated according to standard procedures [45]. Four individuals were studied by species and the results averaged.

### Flower diameter measurements

The floral diameter was measured considering the distance from the most distal part of a given sepal to the most distal part of an opposing petal, in an attempt to capture the maximum diameter of the circle where the flower could be inscribed into. For that, a digital electronic pachymeter (Worker Inc., USA) was used. Flowers with reflexed perianth (e.g. *P. coccinea*, *P. racemosa* etc.), were pressed against a flat surface to spread the sepals and petals to a circular form to get the measurements. At least ten flowers from three unrelated individuals of each species were measured in order to obtain the estimates of the average values and their standard deviation.

### PCR amplification and sequencing

Total DNA was extracted from young leaves dried in silica gel [46], from the same plants used for flow cytometry. Plastid sequences corresponding to the *rbcL* and *rps4* genes, *trnL* intron, and *trnL*–*trnF* intergenic spacer, were amplified using primers and amplification conditions as described before (1F and 1460R primers, [47]; rps45′ and rps43′ primers: [48]; c and d, e and f primers: [49]). PCR products were checked by electrophoresis in 1% agarose gel, stained with Gel Red®, purified with polyethylene glycol 20% [50] and sequenced using the DYEnamic ET Dye Terminator Cycle Sequencing Kit (Amersham Biosciences) in a MegaBACE 1000 automated sequencer (Amersham Biosciences).

### Phylogenetic analyses

The 50 DNA sequences of each gene partition (*rbcL* and *rps4* genes, *trnL* intron, *trnL*-*trnF* intergenic spacer) were aligned separately, visually inspected and manually corrected using the Mega 4.0 software [51]. The sequences were manually merged and the concatenated sequences were submitted to a Bayesian analysis using MrBayes 3.1 [52], [53]. In fact, these sequences are linked by nature, since plastid chromosome is non-recombining, i.e., they are effectively a single locus (with gaps). A substitution model was inferred for each partition using MrModelTest 2.3 [54] to be used in the Bayesian analyses. A total of 10 million generations were run, with a sample frequency of 1000, and 5 million of them were burned out to produce a consensus tree. Thus, we have built a consensus based on 5,000 topologies. This extensive analysis was performed using the CBSU web computing resources (http://cbsuapps.tc.cornell.edu/mrbayes.aspx). A separated tree for each gene partition was also constructed (using its proper model) to compare with the concatenated tree topology. Five million generations were run for each region using the Mr.Bayes, of which 1 million were burned out.

### Comparative Methods

In order to study the relationship between genome sizes and flower diameters of *Passiflora*, it is necessary to take in account that the species share a phylogenetic history, meaning that they are not statistically independent entities. Thus, it is inappropriate the use of standard statistic tests to detect correlations between characteristics of these species. Felsenstein [55] proposed the method of phylogenetically independent contrasts, based on the fact that species themselves are not statistically independent, but the differences between them are. Thus, for each trait (genome size or flower diameter) we subtracted the character values from one another for each terminal species pair and each ancestral node and standardized them (i.e. divided the subtraction by the squared root of the sum of their daughter branch lengths). In order to check whether the branch lengths of the phylogenetic tree adequately standardized the contrasts, we plotted the absolute value of each standardized independent contrast versus its standard deviation (i.e. the square root of the sum of its branch lengths). Any significant linear or nonlinear trend in the plot indicates that the contrasts were not adequately standardized, and thus that the trait values or the branch lengths must be modified. The contrasts values and the branch length values were obtained from the PDTREE program [56], which was also used to estimate the ancestral states of each internal node and their standard deviations for each character. This step requires the re-root of the tree [57]. All these analyses were based on the consensus Bayesian tree (see phylogenetic analyses item).

The standardized contrast values of genome sizes and flower diameters were thus used in correlation inferences in order to detect correlations between them taking the phylogeny into account.

We have also investigated whether the evolution of genome sizes and flower diameters in *Passiflora* followed a random walk (Model A) or a directional change model (Model B) and investigated the tempo and mode of these traits' evolution using kappa (κ), lambda (λ) and delta (δ) parameters. For this, we have used the Continuous option [58], [59] of the BayesTraits program [60]. Table S1 shows the meaning of different values of these parameters, as given in the Continuous manual (http://www.evolution.reading.ac.uk/BayesTraits.html). This program allows the use of a set of different trees to compute the likelihoods associated with different models and parameter values. Thus, we have fed BayesTraits a set of 500 best trees found by MrBayes (representing the last 500,000 generations of the Bayesian inference). Statistical support for the parameter values and model selection were estimated through BayesFactors [54], calculated using the Tracer software [61] based on the harmonic mean of the likelihoods, calculated by the BayesTraits program. To perform these calculations, we run BayesTraits for 100 million generations and applied a burn-in period of 10 million generations. When comparing models using BayesFactors, any positive value favours the dependent model, but conventionally a ratio greater than 2 is taken as positive evidence, greater than 5 is ‘strong’ and greater than 10 is ‘very strong’ evidence.

## Results

Table 1 lists the average genome sizes and flower diameters for the species studied. We found substantial variation of genome sizes (1.073±0.56 pg) and flower diameters (6.12±2.75 cm) within the genus. The range between the largest and smallest genomes is as great as 10x (0.212 pg in *P. organensis*, subgenus *Decaloba*; and 2.208 pg in *P. alata*, subgenus *Passiflora*). In addition, the species presenting the largest GS, *P. alata,* also showed the largest FD (12.52 cm), which is approximately 9x larger than the shortest flower, that of *P. suberosa* (1.42 cm), which has petal-less flowers and belongs to subgenus *Decaloba*. Both GS and FD means were significantly smaller in *Decaloba* when compared to *Passiflora*. In order to determinate the ploidy of the species in study, and to decide if the ploidy level should be considered in our analyses, we have checked chromosome counts for all material used using Feulgen-stained scion root tips (*data not shown*) and all of them showed the reported diploid chromosome numbers.

**Figure 1:**
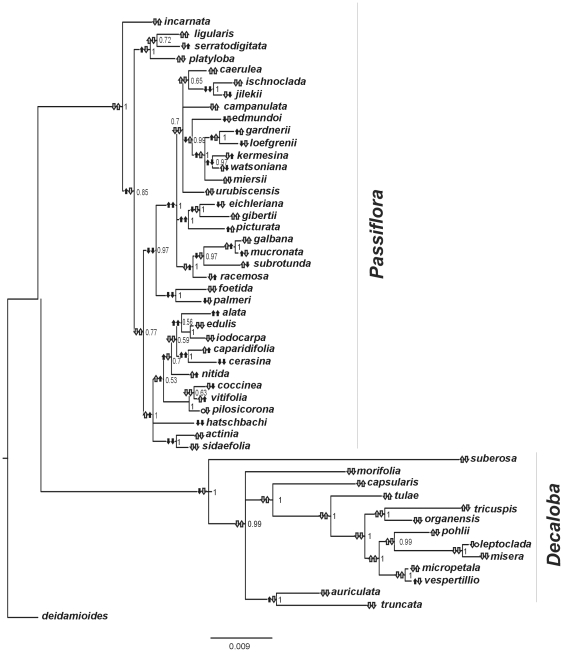
Genome size evolution in *Passiflora*. Bayesian consensus tree based on the concatenated sequences of four chloroplast genes, taking in account the substitution models of each partition. Besides each ancestral node is a fraction number representing its posterior probability. Arrows beside each node represent genome sizes (left) and flower diameters (right). Arrows pointing up mean that there was an increase in average values of GS or FD. Arrows pointing downwards signify a downturn, comparing with the immediately anterior node. Circles represent identical means. Measurements or inferences significantly different than those obtained for the anterior node are represented in black. Measurements not significantly different are represent in white. The inferred values of GS and FD for each ancestral node are shown in Table S3 and Figure S5.

Figure 1 shows the Bayesian consensus phylogeny based on the concatenated sequences (see Table S2 in Supplementary Material to Genbank information about the sequences). This topology is not significantly different from those obtained for the separated genetic regions (there are no conflicting branches with high posterior probabilities greater than 0.95, see supplemental figures S1, S2, S3, S4). Both *Decaloba* and *Passiflora* subgenera are monophyletic with posterior probability (PP) of 1.0. The branching pattern within each subgenus and the different branch lengths among them agree with other studies performed previously [40], [41].

Figure 1 also depicts ranges for the GS and FD for each ancestral and current nodes. Two arrows pointing up or down were used to represent increases or decreases for genome sizes and flower diameters through the tree. Dark arrows represent values significantly different than those inferred for the immediately anterior node, while white arrows represent the difference that was not significant (circles represent identical means). By a significant difference we mean that the mean putative size of a given node is greater or smaller than the size inferred for the anterior node plus or minus its standard error (Table S3 shows the putative values of the ancestral nodes, assigned on Figure S5). For *Decaloba,* we found only three significant modifications in GS, and no significant changes in FD. Conversely, for *Passiflora*, we found several significant modifications, both towards increasing or decreasing for GS and FD.

**Figure 2:**
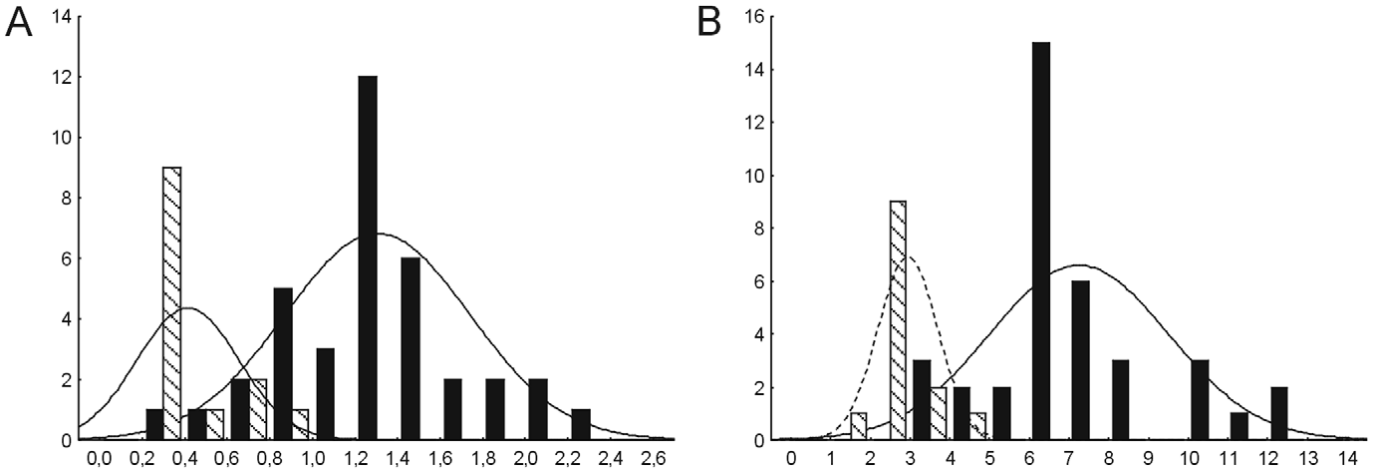
Genome sizes in subgenus *Passiflora* and *Decaloba*. Histograms representing the distribution of (A) genome sizes (expressed as 1C), (B) flower diameters (in cm) within subgenera *Passiflora* (black bars) and *Decaloba* (hachured bars).

Figure 2 shows the histograms with the distributions of current genome sizes and flower diameters of each species within *Passiflora* and *Decaloba* subgenera. *Passiflora* presents significantly larger mean averages and standard deviations for genome sizes and flower diameters (t-tests reveal that the averages and variances are significant different between subgenera for both traits *p*<0.0000001, *data not shown*). The correlation between these values for the whole set of data was high and significant (*r*_GSxFD_ = 0.78, *p*<0.0001). Considering only the species within *Passiflora,* the correlation remained high and significant (*r* = 0.63, *p*<0.0001), but disappeared within *Decaloba* (*r* = −0.38, *p* = 0.196).

**Figure 3:**
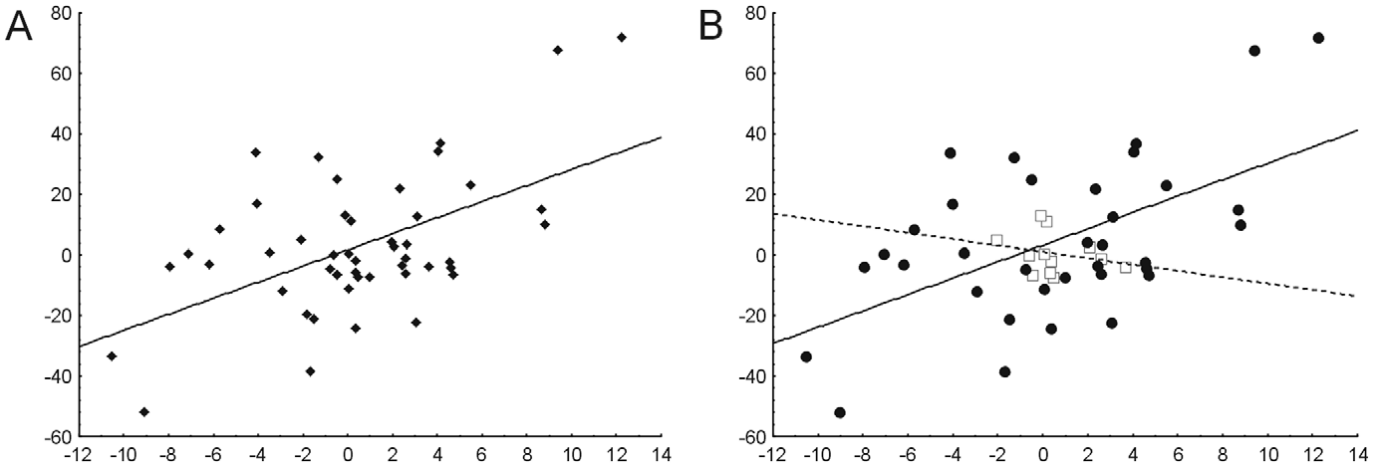
Correlation between genome size and flower diameter in *Passiflora*. Correlation between contrast values of genome sizes (axis X) and flower diameters (axis Y) of (A) complete set of data (*r* = 0.54, *p*<0.0001) and (B) separated by subgenera, (white squares – subgenus *Decaloba*, *r* = 0.25, *p* = 0.44; black circles – subgenus *Passiflora*, *r* = 0.56, *p*<0. 001).

To ensure that these correlations are independent of phylogeny (see material and methods), we have calculated them using the standardized contrast values instead of current values [55]. Preliminary tests showed that the branch lengths of the consensus tree (Figure 1) are appropriated to standardize both GS and FD (data not shown). Figure 3 shows plots of GS X FD standardized contrasts of the total set of data and separated by subgenera. The correlations of the total set, as well as that of *Passiflora* remained significant For *Decaloba*, the lack of significance also remained.

Regarding tempo and mode of GS and FD evolution, the BayesFactor (BF) model B (directional evolution) against model A (random walk model) suggests that these traits did not present any trend toward increases or decreases (BF_GS_ [modelB/modelA] = 0,03; BF_FD_ [modelB/modelA] = 0.584). Table S4 shows the resulting Bayes Factors values calculated for the parameters (lambda, delta and kappa) describing traits (GS and FD) evolution. These tests were performed by the comparison of models in which each parameter is set to 1.0 or 0.0 allowing each parameter to take its maximum likelihood (ML) value. These tests revealed that the parameters lambda (λ) and delta (δ) did not differ from one (1.00) both for GS and FD, as well as the kappa (κ) parameter for GS. On the other hand, the parameter kappa (κ) did not differ from zero (0.00) for FD.

## Discussion

We have investigated the tempo and mode of genome size and flower diameter evolution in 50 species of the *Passiflora* genus, examined if these traits evolve in a random-walk or a directional change models (i.e. if there are any trends towards increases or decreases in these traits) and calculated the correlations between genome sizes (GS) and flower diameters (FD) (Table 1).

Our results revealed that there are no trends towards increases or decreases in GS or FD within the two subgenera of *Passiflora* studied here. The BayesTraits results show that these traits followed a random-walk mode of evolution [59] and thus we used this model in order to infer the trait values of ancestral nodes (Figure 1, Table S3). Indeed, the parameter λ was not significantly different from 1.0 for GS and FD, indicating that the phylogenetic history, as showed in Figure 1, must be considered in order to infer the proper correlation between these traits.

For both FD and GS traits, the values increased and decreased more times within *Passiflora* than within *Decaloba*. Indeed, the subgenus *Passiflora* presents more variance in GS and FD than *Decaloba* (Figure 2). It is important to note that *P. suberosa* (GS = 0,684 ρg), a putative ancient polyploid (2n = 24), which behaves as a diploid, do not present a significant increase in GS compared with those inferred for its immediately ancestral node (GS = 0.67±0.48 ρg).

Either adaptive or neutral theories can explain the differences in variances of GS and FD in *Passiflora* and *Decaloba*. Following the nucleotype effect [35], in an evolutionary (adaptive) scenario in which natural selection favors any DNA loss because it would eventually increase the rate of reproduction, we can infer that as the GS decrease, the likelihood of succeeded small deletions would also decrease, as even small deletions would potentially affect essential DNA sequences. Thus, purifying selection, which avoids the loss of critical sequences, tends to eliminate any selective pressure favoring downsizing genomes, at the same time determining a minimum size for the genome, explaining why the GS within *Decaloba* varied less than within *Passiflora*. An alternative explanation follows the proportional model of GS evolution [19], a strict neutral theory which predicts that large genomes become and remain small more easily than small genomes become and remain large. Also, the rate of genome size evolution is proportional to a given genome size, i.e., the fastest rates occur in the largest genomes.

The ancestral GS inference reveals an initial reduction of GS in *Decaloba*, which was followed by non-significant changes in all nodes; except for the ancestral of *P. auriculada* and *P. truncata* (see Figure 1). Thus, we can argue that an initial reduction restricted the evolution of GS within *Decaloba*. However, the parameter delta, which measures if recent (δ>1) or ancient (δ<1) events differ in importance for the evolution of a trait, does not differ from 1.0 for GS and FD, indicating that, regardless the initial GS reduction in *Decaloba*, the changes on both characters occurred through the evolution of the group.

The kappa (κ) parameter scales branch lengths in tree [59], and can be used to test for a punctuational versus gradual mode of trait evolution. κ = 1.0, as found for GS, denotes a gradual mode of GS evolution, while κ<1.0 compresses longer branches more than shorter ones. In the extreme κ = 0.0, as found for FD, trait evolution is independent of the length of the branch, and is consistent with a punctuational equilibrium model of evolution [59]. These results mean that GS evolutionary rate is more related to the evolutionary rate of the sequences used to construct the tree (neutral plastid sequences) than FD, indicating that FD evolution is driven by natural selection, at least within the subgenus *Passiflora*. Indeed, floral characters (FD included) are in general considered as adaptive traits, which selection is driven by pollinators [62]. In this way, self-compatible species (which is the case of most species within *Decaloba*; [39]) present lower responses to selective pressure than self-incompatible species [63], explaining why FD vary less within *Decaloba* than within *Passiflora*.

Regardless the difference found between the tempo and mode of evolution of GS and FD, we have found a positive and significant correlation between GS and FD within all species, as well as within only subgenus *Passiflora*, considering both current and contrast values (Figure 3), a pattern generally found between other plant organ sizes or other adaptive traits and GS [eg. 21, 23, 27 or 29, but see 30, 31]. High and positive correlations between GS and adaptive traits usually allow the suggestion that variations in genome sizes (GS) should be considered as an adaptive process.

Intriguingly, the positive correlation between GS and FD disappeared when considering only *Decaloba* species. This lack of correlation can be explained by putative constraints in the evolution in both traits, such as the minimum genome size or the proportional evolution for GS, and the lower responses of FD to selection in self-compatible species, as already discussed. Alternatively, we can argue that the correlation between GS and FD is limited by a minimum GS, below which the correlation disappear or became insignificant. Indeed, after the reduction in the basal node of *Decaloba* (node 2, which GS were estimated in 0.67±0.48, see figure S5 and table S2), there were no significant modifications (to increase or decrease) in FD. The only node within the subgenus *Passiflora* which presents a putative GS lower than the node 2 was the node 23 (GS = 0.56±0.18, see figure S5 and table S2), after which no significant changes were found in FD.

Although we found evidence that variations in genome sizes should be considered an adaptive process, we also found clues of limits imposed by a minimum genome size (which could vary across different organisms), which could reduce or even eliminate the possibility of phenotypic responses to environment changes because of reduction of available alternatives of phenotypic expression within the genome. Thus, we suggest that future work involving the study of the evolution of genome sizes take into account a putative size limitation, in order to confirm or overturn our hypothesis.

## Supporting Information

Figure S1 **Bayesian consensus tree based on** ***Passiflora*** **trn-L intron sequences (599 bp).** Besides each ancestral node is a fraction number representing its posterior probability. These sequences were used to build the concatenated tree (Figure 1). The model of choice was the generalized time reversible model (GTR), with the gamma shape parameter alpha = 0.09.
(TIF)

Figure S2 **Bayesian consensus tree based on** ***Passiflora*** **rbcl gene sequences (1348 bp).** Besides each ancestral node is a fraction number representing its posterior probability. These sequences were used to build the concatenated tree (Figure 1). The model of choice was the generalized time reversible model (GTR), with the gamma shape parameter alpha = 0.09 and 65% of invariable sites.
(TIF)

Figure S3 **Bayesian consensus tree based on** ***Passiflora*** **rps4 gene sequences (548 bp).** Besides each ancestral node is a fraction number representing its posterior probability. These sequences were used to build the concatenated tree (Figure 1). The model of choice was the generalized time reversible model (GTR), with the gamma shape parameter alpha = 0.09.
(TIF)

Figure S4 **Bayesian consensus tree based on** ***Passiflora*** **trnLtrnF intergenic spacer sequences (357 bp).** Besides each ancestral node is a fraction number representing its posterior probability. These sequences were used to build the concatenated tree (Figure 1). The model of choice was the Kimura two parameters, with kappa parameter(transitions/transversions) = 2.31 with the gamma shape parameter alpha = 0.1.
(TIF)

Figure S5 **Bayesian consensus tree based on the concatenated sequences of four** ***Passiflora*** **chloroplast genes.** Each ancestral node is identified by a number. The putative ancestral values (mean and standard deviation) of genome sizes (GS, in pg) and flower diameters (FD, in cm) are shown in Table S1.
(TIF)

Table S1 **Parameter values interpretation of the analyses performed in the software Continuous.**
(DOC)

Table S2 **Genbank accession numbers to** ***Passiflora*** **sequences.**
(DOC)

Table S3 ***Passiflora*** **ancestral genome sizes and flower sizes.**
(DOC)

Table S4 **Significance of evolution parameters.**
(DOC)
