# Supplementary figures and images for: Does Variation in Genome Sizes Reflect Adaptive or Neutral Processes? New Clues from Passiflora
Source: PLoS One. 2011 Mar 28;6(3):e18212. doi: 10.1371/journal.pone.0018212 (PMC3065483; doi:10.1371/journal.pone.0018212)

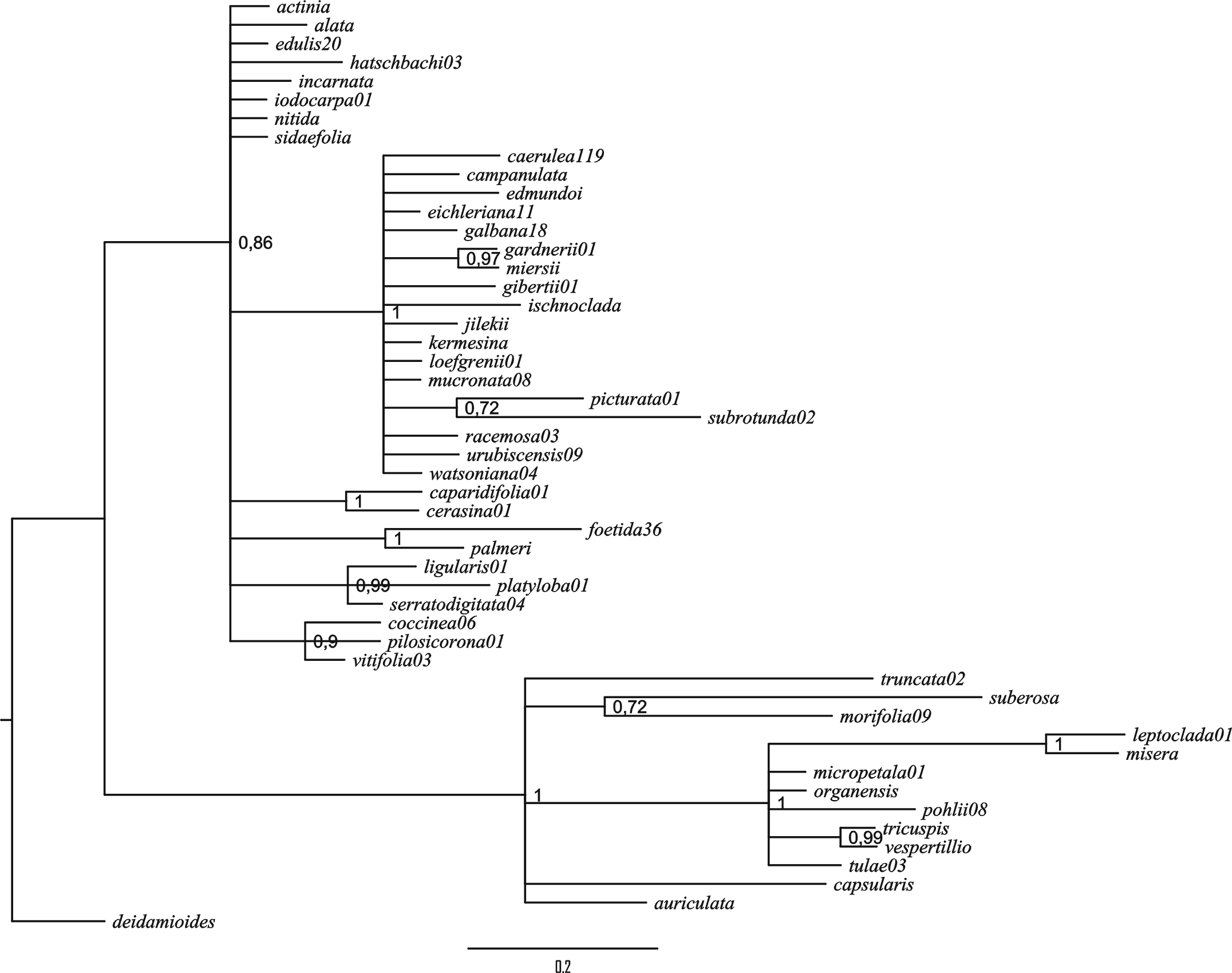

Supplement: Figure S1 — Bayesian consensus tree based on Passiflora trn-L intron sequences (599 bp). Besides each ancestral node is a fraction number representing its posterior probability. These sequences were used to build the concatenated tree (Figure 1). The model of choice was the generalized time reversible model (GTR), with the gamma shape parameter alpha = 0.09. (TIF) [file pone.0018212.s001.tif]

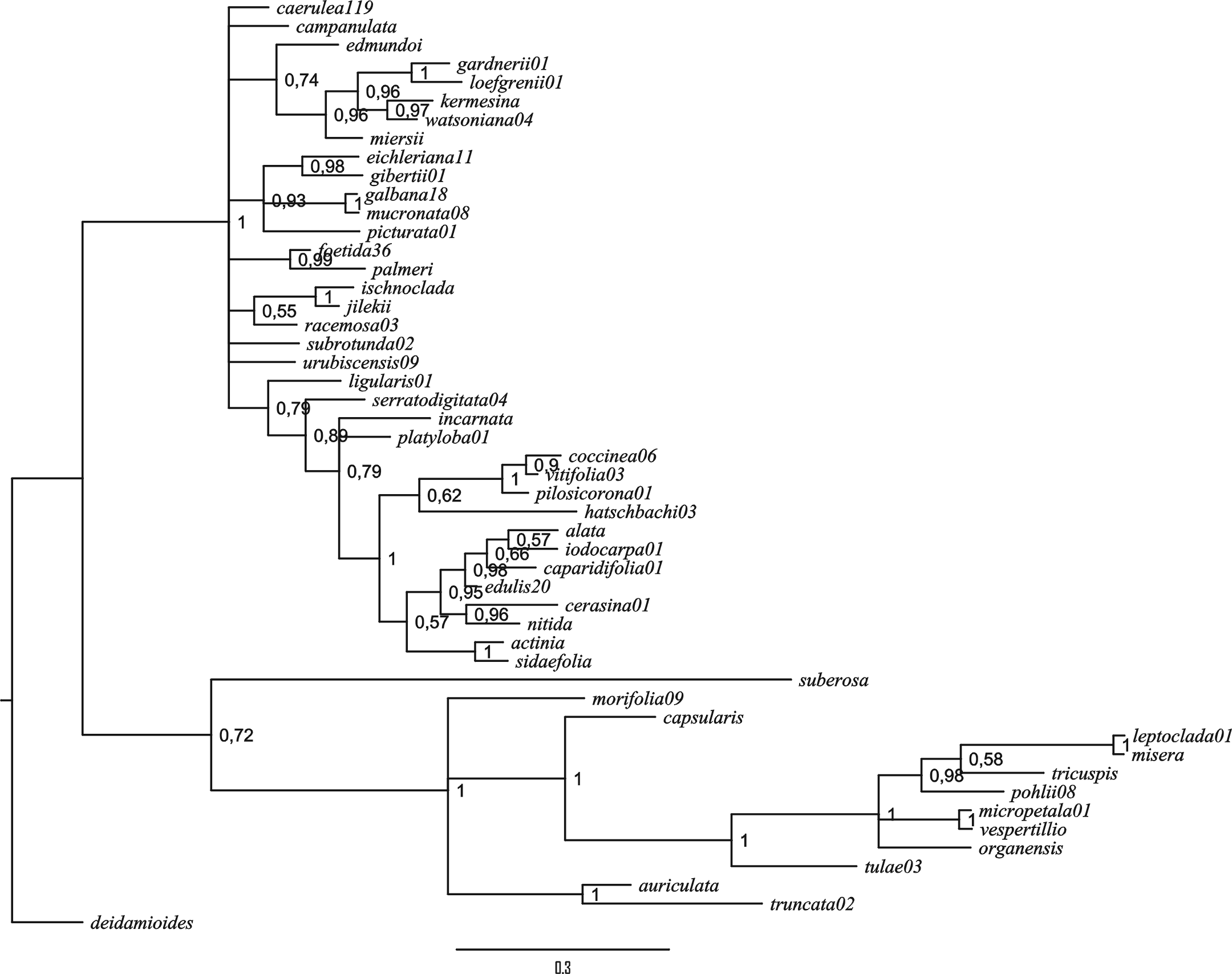

Supplement: Figure S2 — Bayesian consensus tree based on Passiflora rbcl gene sequences (1348 bp). Besides each ancestral node is a fraction number representing its posterior probability. These sequences were used to build the concatenated tree (Figure 1). The model of choice was the generalized time reversible model (GTR), with the gamma shape parameter alpha = 0.09 and 65% of invariable sites. (TIF) [file pone.0018212.s002.tif]

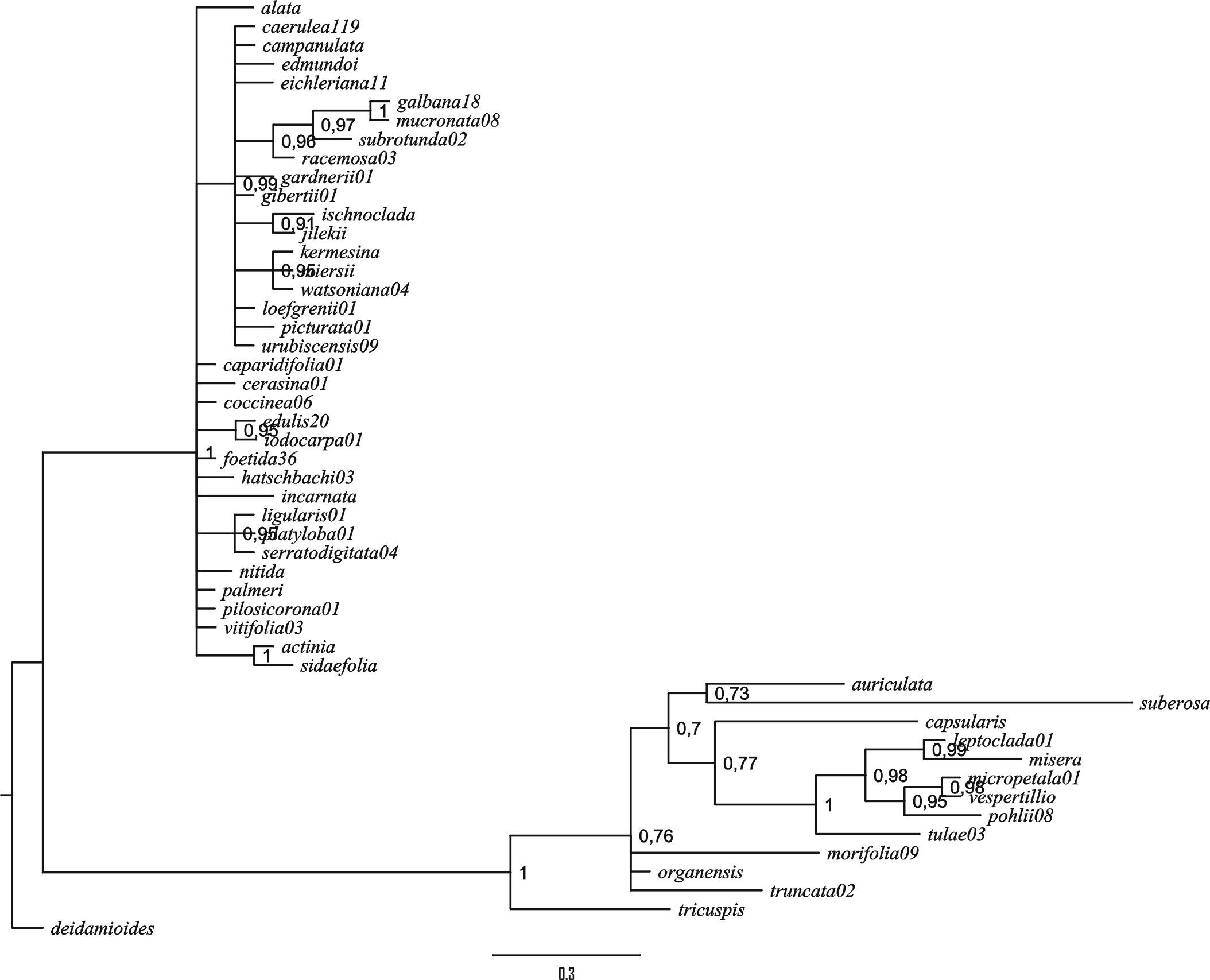

Supplement: Figure S3 — Bayesian consensus tree based on Passiflora rps4 gene sequences (548 bp). Besides each ancestral node is a fraction number representing its posterior probability. These sequences were used to build the concatenated tree (Figure 1). The model of choice was the generalized time reversible model (GTR), with the gamma shape parameter alpha = 0.09. (TIF) [file pone.0018212.s003.tif]

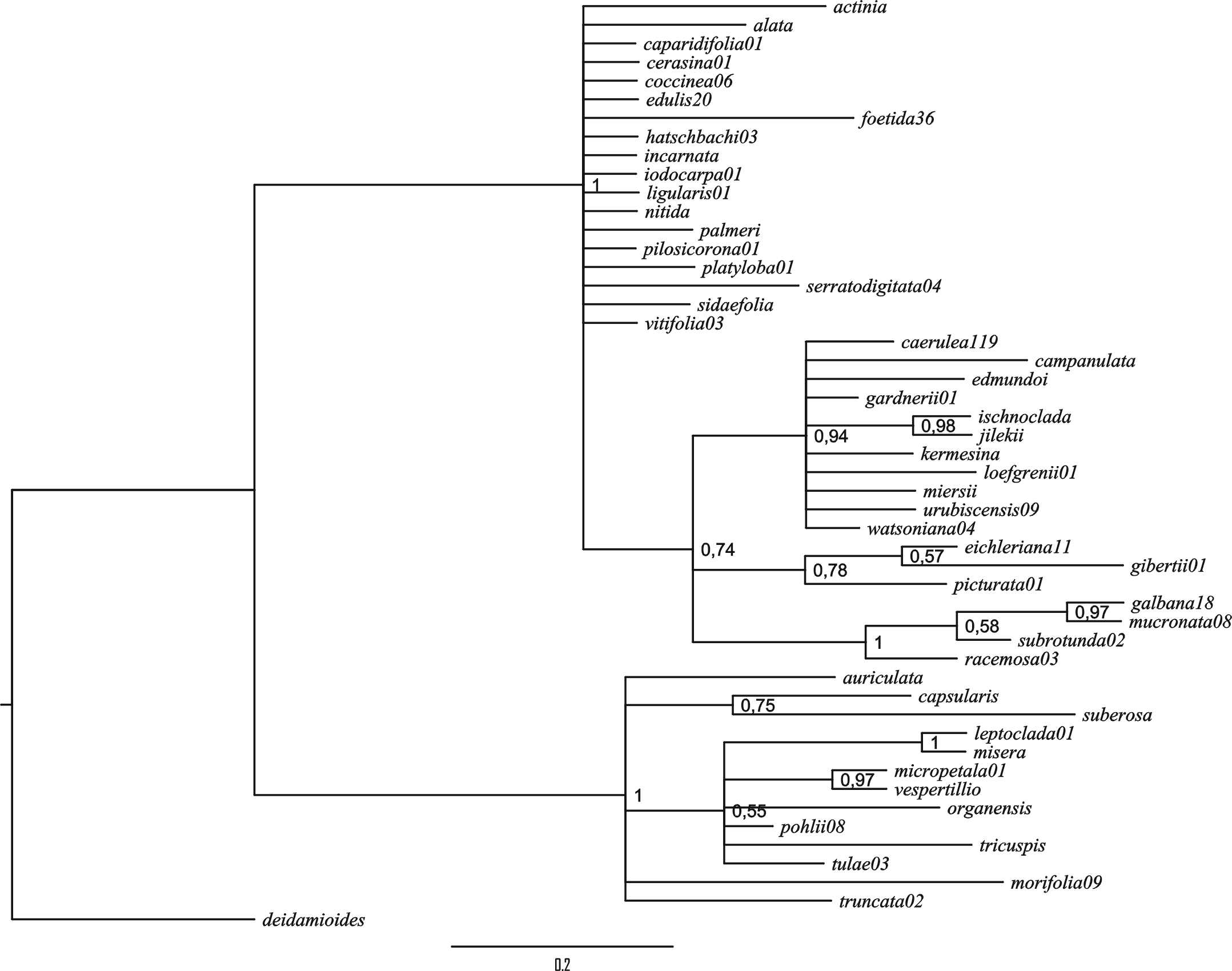

Supplement: Figure S4 — Bayesian consensus tree based on Passiflora trnLtrnF intergenic spacer sequences (357 bp). Besides each ancestral node is a fraction number representing its posterior probability. These sequences were used to build the concatenated tree (Figure 1). The model of choice was the Kimura two parameters, with kappa parameter(transitions/transversions) = 2.31 with the gamma shape parameter alpha = 0.1. (TIF) [file pone.0018212.s004.tif]

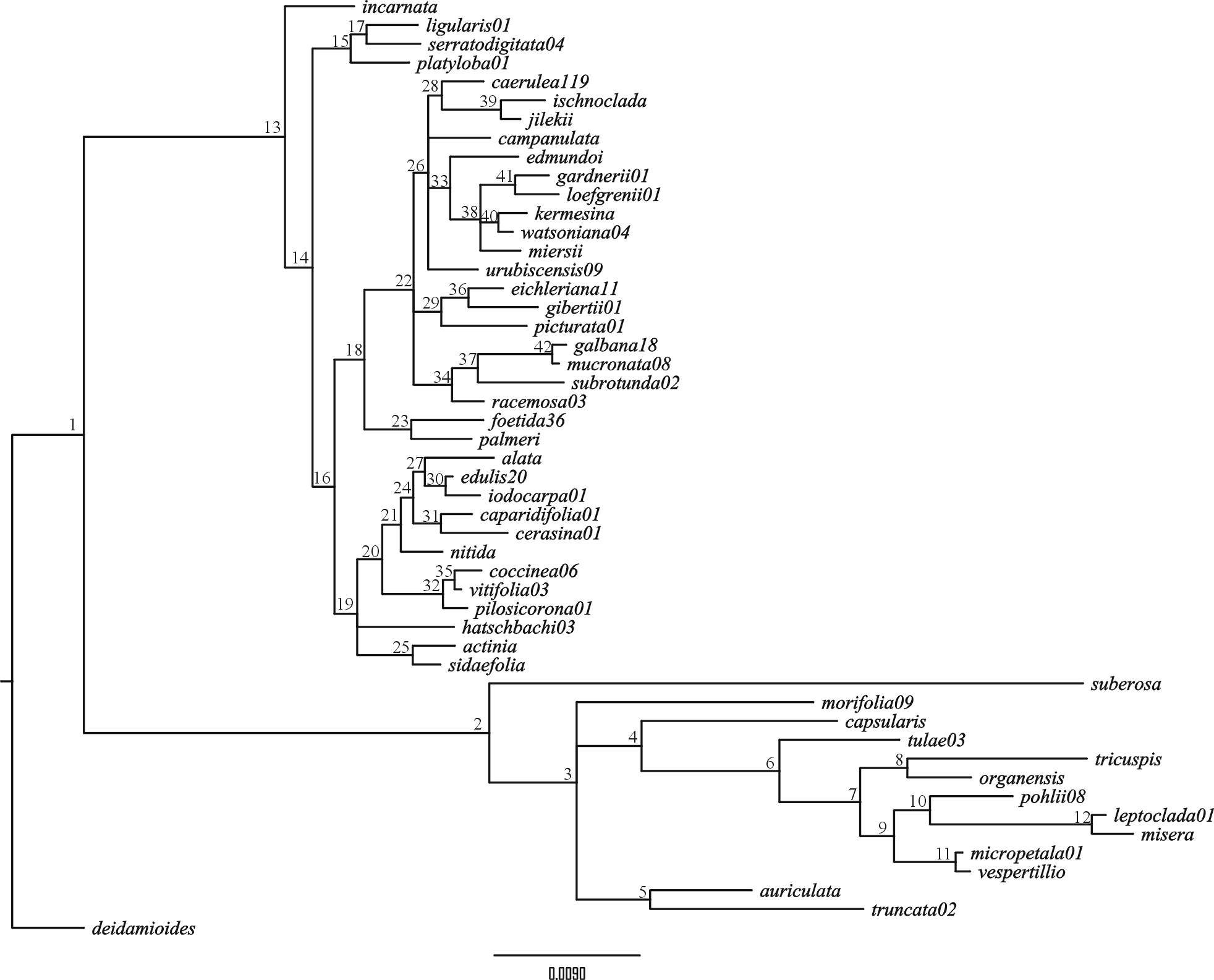

Supplement: Figure S5 — Bayesian consensus tree based on the concatenated sequences of four Passiflora chloroplast genes. Each ancestral node is identified by a number. The putative ancestral values (mean and standard deviation) of genome sizes (GS, in pg) and flower diameters (FD, in cm) are shown in Table S1. (TIF) [file pone.0018212.s005.tif]
