# Supplementary material for: Does Variation in Genome Sizes Reflect Adaptive or Neutral Processes? New Clues from Passiflora
Source: PLoS One. 2011 Mar 28;6(3):e18212. doi: 10.1371/journal.pone.0018212 (PMC3065483; doi:10.1371/journal.pone.0018212)
Supplement: Table S1 — Parameter values interpretation of the analyses performed in the software Continuous. (DOC) [file pone.0018212.s006.doc]

**Table S1. Parameter values interpretation of the analyses performed in the software Continuous.**

| *Parameter* | Action | 0 | <1 | 1 | >1 |
| --- | --- | --- | --- | --- | --- |
| Lambda (λ) | Assess contribution of phylogeny | Star phylogeny  (species independent | Phylogenetic history has minimal effect | Default phylogeny | Not defined |
| kappa (κ) | Scale branch lengths in tree | Punctional evolution | Stasis in longer branches | Default gradualism | Longer branches more change |
| delta (δ) | Scale total path in tree | Not defined | Temporally early change important (adaptive radiation) | Default gradualism | Temporally later change (species-specific adaptation) |
