# Supplementary material for: Does Variation in Genome Sizes Reflect Adaptive or Neutral Processes? New Clues from Passiflora
Source: PLoS One. 2011 Mar 28;6(3):e18212. doi: 10.1371/journal.pone.0018212 (PMC3065483; doi:10.1371/journal.pone.0018212)
Supplement: Table S2 — Genbank accession numbers to Passiflora sequences. (DOC) [file pone.0018212.s007.doc]

**Table S2. Genbank accession numbers to *Passiflora* sequences.**

| Species | RBCL | rps4 | trnL intron | trnL-trnF |
| --- | --- | --- | --- | --- |
| *P. actinia* | HQ900845 | HQ900897 | HQ900949 | HQ901001 |
| *P. alata* | HQ900846 | HQ900898 | HQ900950 | HQ901002 |
| *P. auriculata* | HQ900847 | HQ900899 | HQ900951 | HQ901003 |
| *P. caerulea* | HQ900848 | HQ900900 | HQ900952 | HQ901004 |
| *P. campanulata* | HQ900849 | HQ900901 | HQ900953 | HQ901005 |
| *P. caparidifolia* | HQ900850 | HQ900902 | HQ900954 | HQ901006 |
| *P. capsularis* | HQ900851 | HQ900903 | HQ900955 | HQ901007 |
| *P. cerasina* | HQ900852 | HQ900904 | HQ900956 | HQ901008 |
| *P. cerradensis* | HQ900853 | HQ900905 | HQ900957 | HQ901009 |
| *P. coccinea* | HQ900854 | HQ900906 | HQ900958 | HQ901010 |
| *P. deidamioides* | HQ900855 | HQ900907 | HQ900959 | HQ901011 |
| *P. edmundoii* | HQ900856 | HQ900908 | HQ900960 | HQ901012 |
| *P. edulis* | HQ900857 | HQ900909 | HQ900961 | HQ901013 |
| *P. eichleriana* | HQ900858 | HQ900910 | HQ900962 | HQ901014 |
| *P. foetida* | HQ900859 | HQ900911 | HQ900963 | HQ901015 |
| *P. galbana* | HQ900860 | HQ900912 | HQ900964 | HQ901016 |
| *P. gardnerii* | HQ900861 | HQ900913 | HQ900965 | HQ901017 |
| *P. gibertii* | HQ900862 | HQ900914 | HQ900966 | HQ901018 |
| *P. hatschbachi* | HQ900863 | HQ900915 | HQ900967 | HQ901019 |
| *P. incarnata* | HQ900864 | HQ900916 | HQ900968 | HQ901020 |
| *P. iodocarpa* | HQ900865 | HQ900917 | HQ900969 | HQ901021 |
| *P. ischnoclada* | HQ900866 | HQ900918 | HQ900970 | HQ901022 |
| *P. jilekii* | HQ900867 | HQ900919 | HQ900971 | HQ901023 |
| *P. kermesina* | HQ900868 | HQ900920 | HQ900972 | HQ901024 |
| *P. leptoclada* | HQ900869 | HQ900921 | HQ900973 | HQ901025 |
| *P. ligularis* | HQ900870 | HQ900922 | HQ900974 | HQ901026 |
| *P. loefgrenii* | HQ900871 | HQ900923 | HQ900975 | HQ901027 |
| *P. micropetala* | HQ900872 | HQ900924 | HQ900976 | HQ901028 |
| *P. miersii* | HQ900873 | HQ900925 | HQ900977 | HQ901029 |
| *P. misera* | HQ900874 | HQ900926 | HQ900978 | HQ901030 |
| *P. morifolia* | HQ900875 | HQ900927 | HQ900979 | HQ901031 |
| *P. mucronata* | HQ900876 | HQ900928 | HQ900980 | HQ901032 |
| *P. organensis* | HQ900877 | HQ900929 | HQ900981 | HQ901033 |
| *P. nitida* | HQ900878 | HQ900930 | HQ900982 | HQ901034 |
| *P. palmeri* | HQ900879 | HQ900931 | HQ900983 | HQ901035 |
| *P. picturata* | HQ900880 | HQ900932 | HQ900984 | HQ901036 |
| *P. pilosicorona* | HQ900881 | HQ900933 | HQ900985 | HQ901037 |
| *P. platyloba* | HQ900882 | HQ900934 | HQ900986 | HQ901038 |
| *P. pohlii* | HQ900883 | HQ900935 | HQ900987 | HQ901039 |
| *P. racemosa* | HQ900884 | HQ900936 | HQ900988 | HQ901040 |
| *P. serratodigitata* | HQ900885 | HQ900937 | HQ900989 | HQ901041 |
| *P. sidaefolia* | HQ900886 | HQ900938 | HQ900990 | HQ901042 |
| *P. suberosa* | HQ900888 | HQ900940 | HQ900992 | HQ901044 |
| *P. subrotunda* | HQ900889 | HQ900941 | HQ900993 | HQ901045 |
| *P. tricuspis* | HQ900890 | HQ900942 | HQ900994 | HQ901046 |
| *P. truncata* | HQ900891 | HQ900943 | HQ900995 | HQ901047 |
| *P. tulae* | HQ900892 | HQ900944 | HQ900996 | HQ901048 |
| *P. urubiscensis* | HQ900893 | HQ900945 | HQ900997 | HQ901049 |
| *P. vespertillio* | HQ900894 | HQ900946 | HQ900998 | HQ901050 |
| *P. vitifolia* | HQ900895 | HQ900947 | HQ900999 | HQ901051 |
| *P. watsoniana* | HQ900896 | HQ900948 | HQ901000 | HQ901052 |
