# Supplementary material for: Does Variation in Genome Sizes Reflect Adaptive or Neutral Processes? New Clues from Passiflora
Source: PLoS One. 2011 Mar 28;6(3):e18212. doi: 10.1371/journal.pone.0018212 (PMC3065483; doi:10.1371/journal.pone.0018212)
Supplement: Table S3 — Passiflora ancestral genome sizes and flower sizes. (DOC) [file pone.0018212.s008.doc]

**Table S3. *Passiflora* ancestral genome sizes and flower sizes.**

Estimations were calculated for the 42 ancestral nodes highlighted in Figure S5.

| ***Node*** | ***GS*** | ***FD*** | ***Node*** | ***GS*** | ***FD*** |
| --- | --- | --- | --- | --- | --- |
| **1** | 0.96 (0.33) | 5.87 (1.60) | **22** | 1.28 (0.12) | 6.57 (0.61) |
| **2** | 0.67 (0.48) | 2.31 (2.34) | **23** | 0.56 (0.18) | 4.11 (0.88) |
| **3** | 0.56 (0.31) | 2.99 (1.51) | **24** | 1.66 (0.15) | 9.95 (0.72) |
| **4** | 0.40 (0.29) | 3.01 (1.42) | **25** | 1.04 (0.14) | 6.41 (0.67) |
| **5** | 0.88 (0.22) | 2.89 (1.10) | **26** | 1.27 (0.13) | 6.30 (0.62) |
| **6** | 0.31 (0.23) | 3.99 (1.16) | **27** | 1.89 (0.16) | 10.80 (0.8) |
| **7** | 0.27 (0.22) | 3.09 (1.07) | **28** | 1.32 (0.13) | 6.27 (0.66) |
| **8** | 0.27 (0.26) | 2.83 (1.30) | **29** | 1.82 (0.18) | 7.47 (0.91) |
| **9** | 0.28 (0.18) | 3.56 (0.88) | **30** | 1.30 (0.06) | 7.20 (0.31) |
| **10** | 0.29 (0.19) | 2.75 (0.96) | **31** | 1.89 (0.12) | 11.60 (1.60) |
| **11** | 0.26 (0.06) | 3.89 (0.30) | **32** | 1.40 (0.1) | 10.96 (0.52) |
| **12** | 0.26 (0.09) | 2.75 (0.44) | **33** | 1.00 (0.17) | 6.40 (0.81) |
| **13** | 0.82 (0.18) | 6.77 (0.88) | **34** | 1.15 (0.12) | 8.10 (0.61) |
| **14** | 1.25 (0.18) | 6.70 (0.86) | **35** | 1.37 (0.10) | 10.73 (0.50) |
| **15** | 1.50 (0.16) | 6.09 (0.78) | **36** | 1.56 (0.17) | 6.80 (0.93) |
| **16** | 1.16 (0.16) | 7.25 (0.77) | **37** | 1.30 (0.19) | 5.96 (0.93 |
| **17** | 1.39 (0.15) | 6.30 (0.74) | **38** | 1.41 (0.13) | 6.52 (0.63) |
| **18** | 0.83 (0.19) | 5.47 (0.93) | **39** | 0.94 (0.14) | 5.13 (0.67) |
| **19** | 1.30 (0.15) | 8.30 (0.76) | **40** | 1.27 (0.11) | 7.24 (0.54) |
| **20** | 1.40 (0.17) | 9.99 (0.85) | **41** | 1.73 (0.12) | 6.85 (0.61) |
| **21** | 1.71 (0.13) | 9.93 (0.66) | **42** | 1.42 (0.08) | 7.11 (0.38) |
