# Supplementary material for: Does Variation in Genome Sizes Reflect Adaptive or Neutral Processes? New Clues from Passiflora
Source: PLoS One. 2011 Mar 28;6(3):e18212. doi: 10.1371/journal.pone.0018212 (PMC3065483; doi:10.1371/journal.pone.0018212)
Supplement: Table S4 — Significance of evolution parameters. (DOC) [file pone.0018212.s009.doc]

**Table S4. Significance of evolution parameters.**

Bayes Factors for testing whether the ML estimated values (dependent model) of the parameters lambda (λ), delta (δ) and kappa (κ) of the evolution of genome sizes (GS) and flower diameters (FD) were significant better than fixed values of these parameters set as zero (0.0) or one (1.0). The meanings of parameters values are listed in Table S1. An asterisk (*) indicates that a given ML value is significantly better than the fixed value. Conventionally, values greater than 2 are taken as positive evidence, greater than 5 are ‘strong’ and greater than 10 are ‘very strong’ evidence.

| Parameters | Dependent Model  (ML value) | Value = 0 | Value = 1 | Value considered |
| --- | --- | --- | --- | --- |
| λ(GS) | 0.919 | 5E08 * | 0.027 | 1.00 |
| δ(GS) | 0.551 | N/A | 0.701 | 1.00 |
| κ(GS) | 0.566 | 3.098 * | 0.857 | 1.00 |
| λ(FD) | 0.868 | 3E10* | 0.441 | 1.00 |
| δ(FD) | 0.448 | N/A | 0,64 | 1.00 |
| κ(FD) | 0.256 | 1.87 | 88.513* | 0.00 |
